# Acidification of endothelial Weibel-Palade bodies is mediated by the vacuolar-type H^+^-ATPase

**DOI:** 10.1371/journal.pone.0270299

**Published:** 2022-06-29

**Authors:** Julian Terglane, Dirk Menche, Volker Gerke

**Affiliations:** 1 Institute of Medical Biochemistry, Center for Molecular Biology of Inflammation, University of Muenster, Muenster, Germany; 2 Kekulé Institute of Organic Chemistry and Biochemistry, University of Bonn, Bonn, Germany; National Cerebral and Cardiovascular Center, JAPAN

## Abstract

Weibel-Palade bodies (WPB) are unique secretory granules of endothelial cells that store the procoagulant von-Willebrand factor (VWF) in a highly compacted form. Upon exocytosis the densely packed VWF unfurls into long strands that expose binding sites for circulating platelets and thereby initiate the formation of a platelet plug at sites of blood vessel injury. Dense packing of VWF requires the establishment of an acidic pH in the lumen of maturing WPB but the mechanism responsible for this acidification has not yet been fully established. We show here that subunits of the vacuolar-type H^+^-ATPase are present on mature WPB and that interference with the proton pump activity of the ATPase employing inhibitors of different chemical nature blocks a reduction in the relative internal pH of WPB. Furthermore, depletion of the V-ATPase subunit V0d1 from primary endothelial cells prevents WPB pH reduction and the establishment of an elongated morphology of WPB that is dictated by the densely packed VWF tubules. Thus, the vacuolar-type H^+^-ATPase present on WPB is required for proper acidification and maturation of the organelle.

## Introduction

Endothelial cells regulate vascular hemostasis by supplying the blood with factors promoting and also resolving coagulation. Among them, von Willebrand factor (VWF) is the most prominent example. VWF is acutely released from endothelial cells in response to blood vessel injury and then initiates the primary hemostatic response both, by binding to exposed collagens of the subendothelial matrix and capturing circulating platelets that eventually from a platelet plug at the site of injury. To acutely respond to vascular injury, VWF is stored in a ready-to-be-released form in endothelial-specific storage granules, the Weibel-Palade bodies (WPB). WPB are characterized by a unique, rod-like shape and they acquire this shape as a result of VWF multimerization, tubulation and dense packing inside the organelle (for reviews see [1–4]).

VWF is synthesized as a preproprotein at the ER with the signal peptide cleaved off following synthesis. In the ER and Golgi further maturation occurs and VWF forms long concatamers linked by C-terminal and N-terminal disulfide bridges. Defined numbers of VWF molecules are loaded into ministack Golgi cisternae and eventually emerge as immature WPB. WPB are then transported to the cell periphery along microtubule tracks and acquire additional components from the endosomal system, thereby sharing characteristics with lysosomes-related organelles. This similarity also includes the association of a specific RabGTPase, Rab27a, that is recruited from the cytoplasm and serves, among other things, as a link to the cortical actin cytoskeleton via the Rab27a effector MyRIP (for reviews see [1–3, 5, 6]). During the entire process of WPB maturation, the VWF multimers are further condensed assisted by the formation of helical tubules, eventually resulting in WPB that contain densily packed, paracrystalline VWF tubules enwrapped in a tight-fitting membrane. The tightly packed VWF tubules thereby define the unique structure of WPB as long rod-shaped organelles, 1–5 μm in length and approximately 200 nm in diameter (for review see [2]). Critical for the assembly of VWF into helical tubules are elevated Ca^2+^ concentrations and a low pH of appr. 5.5 in the mature WPB [7–11]. The pH dependence of this VWF packing allows VWF multimers to rapidly unfurl once exocytic fusion of WPB with the plasma membrane leads to neutralization [12]. Thus, as in many other processes, a pH switch regulates the transition from an inactive (storage) form, here the paracrystalline VWF tubules, to the physiologically active variant, the long VWF strings that unfold in the blood stream and thereby expose binding sites for glycoprotein Ib and other receptors on activated platelets [12–17]. The physiological relevance of this pH-dependent spring-loaded VWF packing and the subsequent rapid release of long VWF multimers is evident, for example, in von-Willebrand disease, the most common inherited bleeding disorder, in which VWF levels, packing and/or secretion are compromised due to mutations, in most cases in the VWF gene itself (for reviews see [18, 19]).

Despite the crucial importance of the low pH inside WPB for proper VWF packing and thus the correct release of VWF multimers in the control of vascular hemostasis, the molecular mechanism(s) underlying acidification in maturing WPB have not been fully resolved. Most likely, ATPase-mediated proton pumping is required and the presence of the V0a1, V0a2, V0d1 and V1A subunits of the vacuolar-type H^+^-ATPase (V-ATPase) on WPB has recently been reported [20–22]. The V-ATPase, a multi-subunit complex responsible for the acidification of compartments, comprises a membrane-spanning V0 sector composed of the subunits a, c, c”, d and e, and a cytosolic V1 sector harboring the subunits A, B, C, D, E, F, G, H [23–25]. Thus, to address the molecular basis of WPB acidification, we attempted to directly inhibit V-ATPase activity and asses the effects on WPB maturation. In line with the recent identification of several subunits of the V-ATPase, we now show that the cytosolic V1G1 subunit of the V-ATPase complex also localizes to WPB. Importantly, we also show that V-ATPase activity is responsible for WPB acidification as inhibition of the proton pump activity of the enzyme and knockdown of a specific V-ATPase subunit interfere with WPB acidification and hence the formation of mature rod-shaped granules.

## Results and discussion

### V-ATPase subunits localize to endothelial WPB

**Fig 1:**
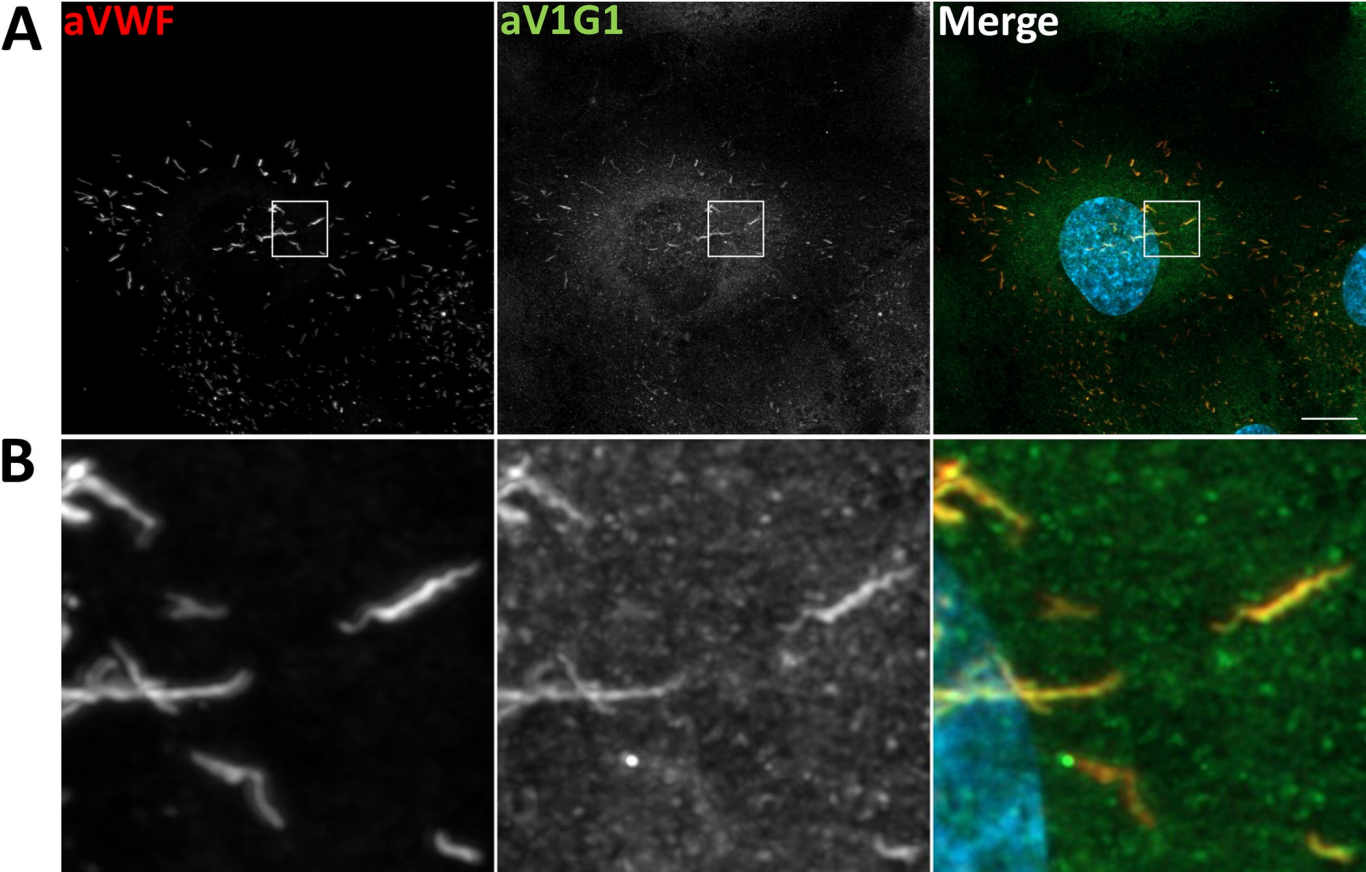
The V-ATPase subunit V1G1 localizes to WPB. **A.** HUVEC were cultivated on collagen coated coverslips until they reached confluency, then fixed and stained for VWF as a general WPB marker (red) and for ATP6V1G1 (green) using the respective antibodies. Shown are confocal microscope images displaying maximum intensity projections of z-stacks. Boxed area is enlarged in **B**. DAPI stainings are shown in blue. Scale bars: 10 μm.

Our previous proximity proteomic approach employing the WPB-associated GTPases Rab27a and Rab3b as baits identified several V-ATPase subunits most likely residing in mature WPB. Specifically, the subunits V0a2 and V0d1 were found enriched in both, the Rab27a and Rab3b associated proteome [21]. Subunits V0a1 and V0d1 were also present in subcellular fractions enriched in WPB that were prepared by density gradient fractionation [20]. Moreover, the V0a1, V0a2, V0c and V1A subunits were recently localized to mature and nascent WPB, respectively, and V0a1 was reported to play a role in the separation of WPB from the trans-Golgi network (TGN) [22]. Because these studies mainly reported the presence of subunits of the V0 subcomplex, i.e. the transmembrane portion of the V-ATPase, and both, the V0 and the cytoplasmic V1 subcomplex are required to form an active enzyme, we first aimed at directly localizing a V1 subunit to endothelial WPB by employing microscopic analyses. Therefore, we used subunit specific antibodies in immunofluorescence approaches which revealed a partial localization of V1G1 to WPB identified by co-staining with anti-VWF antibodies (Fig 1). Interestingly, it appears that not all WPB are positive for V1G1 staining with low or even no V1G1 immunoreactivity in particular on less elongated WPB. This could reflect a lower abundance of this V-ATPase subunit on less mature, i.e. less elongated, WPB but could also be due to a limited sensitivity of the immunofluorescence approach.

Together with the identification of V0 subunits in the proteomic approaches, these data indicate that both, the membrane-spanning as well as the cytoplasmic subcomplex of the V-ATPase are present on endothelial WPB most likely forming an active enzyme. Due to the lack of appropriate antibodies and the very low expression levels of several V-ATPase subunit constructs in HUVEC, we could not analyze whether all subunits of the full enzyme complex are present on WPB. Likewise, the proteomic approaches only identified a few subunits as WPB-associated. This could be due to the inherent difficulty of obtaining sufficient peptides from membrane proteins in such approaches. However, in line with the weak antibody signals observed in our immunofluorescence assays this could also reflect a rather low copy number of V-ATPase complexes per WPB.

### V-ATPase activity is required for WPB acidification

**Fig 2:**
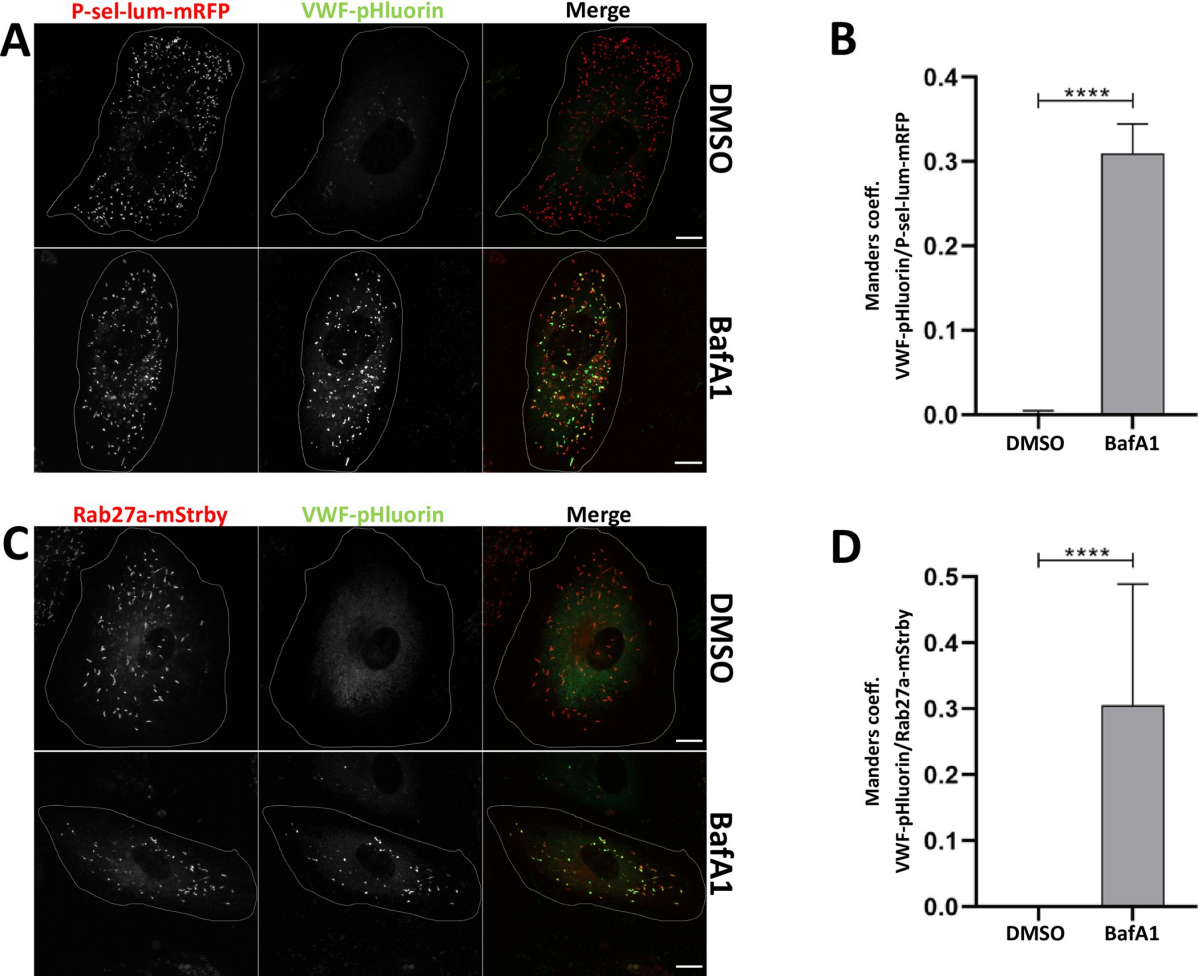
Acidification of WPB is inhibited by bafilomycin A1. **A.** Colocalization of P-sel-lum-mRFP and VWF-pHluorin fluorescence signals in HUVEC treated with the V-ATPase-specific inhibitor bafilomycin A1. HUVEC cotransfected with P-sel-lum-mRFP and VWF-pHluorin were incubated with 0.1% DMSO (upper panel) or 250 nM BafA1 (lower panel), respectively, at 37°C for 2 h and subsequently subjected to live cell imaging. Shown are images of maximum intensity projections of z-stacks. Cell circumferences are indicated by lines. Scale bars: 10 μm. **B.** The degree of colocalization of the fluorescence signals was quantified by determining Manders coefficient 1 which corresponds to the fraction of P-sel-lum-mRFP overlapping with VWF-pHluorin. n = 30. Bars indicate the median. Error bars show 95% confidence interval. Statistics were conducted using a Mann-Whitney test. ****p≤ 0.0001, ***p≤ 0.001, **p≤0.01, *p≤0.05. **C.** Rab27a-positive WPB are affected by bafilomycin A1 treatment. Cells expressing Rab27a-mStrawberry and VWF-pHluorin were incubated with 0.1% DMSO (upper panel) or 250 nM BafA1 (lower panel), respectively, at 37°C for 2 h and subsequently analyzed via live cell imaging. Shown are images of maximum intensity projections of z-stacks. Cell circumferences are indicated by lines. Scale bars: 10 μm. **D.** The extent of colocalization of the fluorescence signals was quantified by determining Manders coefficient 1 which corresponds to the fraction of Rab27a-mStrawberry overlapping with VWF-pHluorin. n = 10. Bars indicate the median. Error bars show 95% confidence interval. Statistics were conducted using a Mann-Whitney test. ****p≤ 0.0001, ***p≤ 0.001, **p≤0.01, *p≤0.05.

Given the presence of V-ATPase subunits of both subcomplexes on WPB we next attempted to determine whether the proton pump activity is responsible for WPB acidification. Therefore, we developed a microscopy-based assay as a means to qualitatively reveal luminal pH changes in WPB. HUVEC were transfected with constructs encoding the luminal domain of P-selectin coupled to RFP (as a general luminal WPB marker) and VWF-pHluorin (as a luminal protein showing pH sensitive fluorescence intensity). Western blot analysis revealed that both constructs remained largely intact following their ectopic expression (S1 Fig). Mature WPB exhibit a luminal pH of appr. 5.5, conditions resulting in significantly reduced pHluorin fluorescence intensity. Neutralization of the luminal pH typically occurs upon exocytotic fusion with the plasma membrane and markedly increases the pHluorin fluorescence intensity, which allows the recording of individual WPB-PM fusion events as bright fusion spots (see, for example [26, 27]). However, a VWF-pHluorin fluorescence increase inside intracellular WPB was only observed when the base NH_4_Cl was applied to HUVEC to artificially quench the luminal low pH of acidic organelles such as WPB (S2 Fig). Hence, VWF-pHluorin residing in mature WPB can be used to record pH changes inside the (P-sel-lum-mRFP positive) organelle. We next applied a well-estalished V-ATPase inhibitor, the lactone bafilomycin A1 (BafA1), to HUVEC expressing VWF-pHluorin and P-sel-lum-mRFP. Fig 2A shows that BafA1 treatment induces a strong pHluorin signal in the majority of P-sel-lum-mRFP positive WPB which remain non-visible in the pHluorin channel in vehicle-treated HUVEC. In the latter case the more general and weaker VWF-pHluorin fluorescence most likely reflects ER localization of the construct. To quantify the BafA1 effect and compare the WPB-specific intensity increase of the pHluorin fluorescence signal in BafA1 and control vehicle treated cells we measured the extent of colocalization of the RFP and pHluorin signals as the latter is only observed upon neutralization of the luminal WPB pH. This analysis revealed a marked increase of signal colocalization as a result of BafA1 treatment (Fig 2B). Following transient transfection, expression of the two different constructs used in the assay, P-sel-lum-mRFP and VWF-pHluorin, could vary among cells possibly compromising the colocalization data. Therefore, we also generated a WPB marker construct, in which pHluorin and mRFP were fused in tandem to VWF (VWF-pHluorin-mRFP), and subjected HUVEC expressing this construct to BafA1 treatment. In line with the approach using two different constructs (P-sel-lum-RFP and VWF-pHluorin), this experiment shows that the RFP-positive WPB are hardly visible in the pHluorin channel in control conditions whereas BafA1 treatment induces a significant increase in the pHluorin fluorescence inside WPB, thereby elevating the colocalization coefficient (S3 Fig). Thus, expression of the two different WPB marker constructs P-sel-lum-RFP and VWF-pHluorin faithfully mimics the behaviour of a single tandem construct (VWF-pHluorin-mRFP) when analyzing the pH-dependent changes of pHluorin fluorescence inside WPB.

To assess whether the mature and more acidic WPB are particularly affected by the BafA1 treatment we also performed a colocalization analysis of the fluorescence signals of VWF-pHluorin and Rab27a-RFP, which served as a well-established marker for the mature, more peripherally localized WPB. Again, BafA1 treatment lead to an increase in pHluorin fluorescence, which is almost exclusively seen in Rab27a-positive WPB, and consequently a significantly elevated colocalization of the fluorescence signals of the two markers is observed upon BafA1 treatment (Fig 2C and 2D). Thus, acidification of WPB which is required for the tight packing of VWF tubules inside the organelle is likely driven by a BafA1-sensitive proton pump.

**Fig 3:**
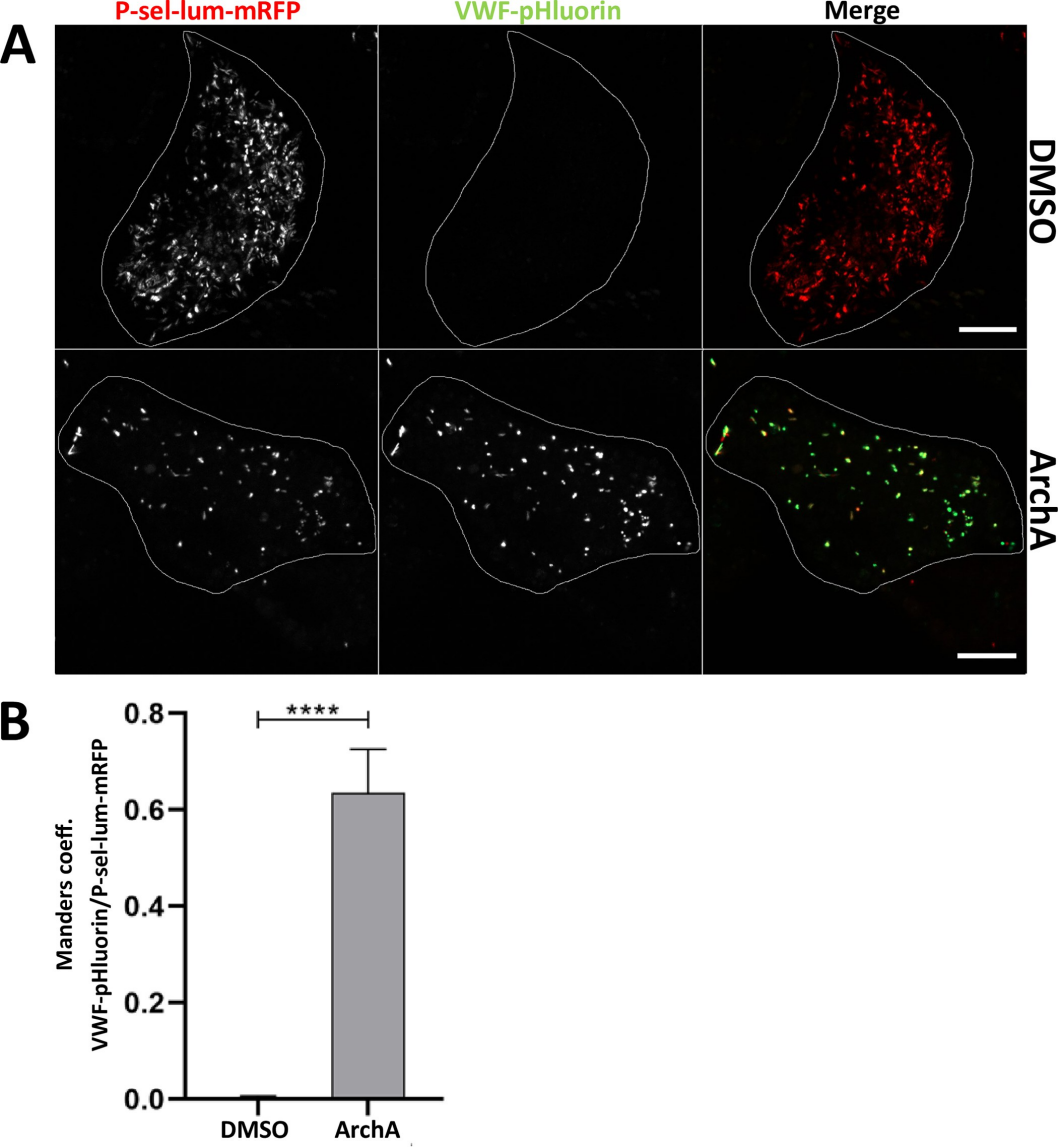
The V-ATPase-specific inhibitor archazolid A interferes with WPB acidification. A. HUVEC expressing P-sel-lum-mRFP and VWF-pHluorin were incubated with 0.02% DMSO (upper panel) or 2 nM ArchA (lower panel), respectively, at 37°C for 24 h and subsequently analyzed via live cell microscopy. Shown are images of maximum intensity projections of z-stacks. Cell circumferences are indicated by lines. Scale bars: 10 μm. B. The degree of colocalization of the mRFP and pHluorin fluorescence signals was quantified by determining Manders coefficient 1 which corresponds to the fraction of P-sel-lum-mRFP overlapping with VWF-pHluorin. n = 30. Bars indicate the median. Error bars show 95% confidence interval. Statistics were conducted using a Mann-Whitney test. ****p≤ 0.0001, ***p≤ 0.001, **p≤0.01, *p≤0.05.

**Fig 4:**
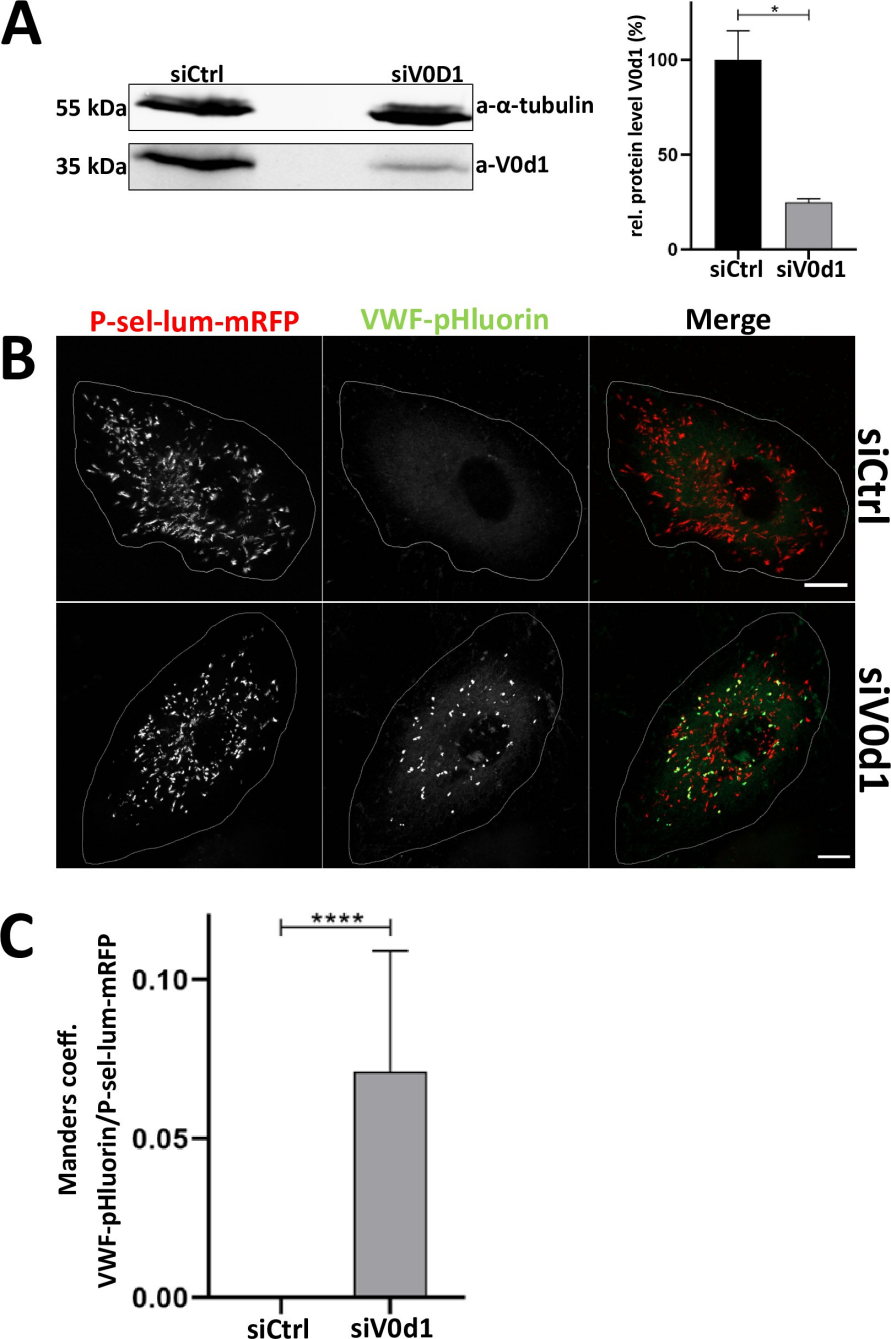
Knockdown of the V-ATPase subunit V0d1 prevents acidification of WPB. HUVEC were transfected with the respective siRNA, P-sel-lum-mRFP and VWF-pHluorin. **A.** 24 h later cells were lysed and subjected to Western blot analysis with anti-V0d1 antibodies. Probing with anti-tubulin antibodies served as loading control. One representative blot is shown on the left. Right, V0d1 band intensities of 3 blots were measured and normalized to intensities of the loading control. Mean of the normalized intensity of the siControl samples was set to 100%. Bars indicate mean and error bars show standard deviation. Statistics were conducted using an unpaired t-test with Welch´s correction. ****p≤ 0.0001, ***p≤ 0.001, **p≤0.01, *p≤0.05. **B-C.** 24 h post transfection, cells were analyzed via live cell microscopy (B) and the degree of colocalization of the mRFP and pHluorin fluorescence signals was quantified by calculating Manders coefficient 1 which corresponds to the fraction of P-sel-lum-mRFP overlapping with VWF-pHluorin (C). Shown are images of maximum intensity projections of z-stacks. Cell circumferences are indicated by lines. Scale bars: 10 μm. n = 30. Bars indicate the median. Error bars show 95% confidence interval. Statistics were conducted using a Mann-Whitney test. ****p≤ 0.0001, ***p≤ 0.001, **p≤0.01, *p≤0.05.

To corroborate these data we employed another chemically different V-ATPase inhibitor, the polyketide archazolid A [28–30]. Similar to BafA1, archazolid A treatment led to a marked neutralization of WPB as visualized by the increase in VWF-pHluorin fluorescence and quantified by the high degree of colocalization of the VWF-pHluorin and P-sel-lum-mRFP fluorescence signals (Fig 3). To strengthen the findings of the chemical inhibitor experiments we also attempted to inhibit V-ATPase activity by depleting an individual subunit. As assembly of the full V-ATPase complex is required for proper proton pumping activity (for review see [25, 31]), we rationalized that depletion of one of the subunits would interfere significantly with V-ATPase mediated acidifications. Because the V0d1 subunit had been identified in the two proteomic WPB screens carried out so far [20, 21], we chose to target this subunit by siRNA-mediated knockdown. Furthermore, downregulation of V0d1 in LLC-MK2 cells has recently been shown to interfere with proper acidification of lysosomes, which requires V-ATPase activity [32]. Fig 4A shows that a significant downregulation to approximately 20% of the initial protein level could be achieved in the primary HUVEC. Cells depleted of V0d1 were then subjected to analysis of the VWF-pHluorin/P-sel-lum-mRFP signal colocalization. This revealed a lack of WPB acidification in the siV0d1 as compared to control siRNA treated cells (Fig 4B and 4C). Interestingly, a recent study identified the Hermansky-Pudlak syndrome protein HPS6 as an interaction partner of the V0d1 subunit implicated in mediating a transport of this subunit to WPB. Moreover, depletion of V0d1 in this study resulted in morphological WPB alterations (less elongated) [33] similar to those observed upon pharmacological V-ATPase inhibition (Figs 2 and 3). Thus, inhibition of the proton pump activity of the V-ATPase by pharmacologically targeting a subunit of the V0 subcomplex as well as specific depletion of one of the V0 subunits interferes with WPB acidification and most likely also with proper VWF (and WPB) maturation. Interestingly it was shown before that elevation of the acidic luminal pH of WPB had no effect on the regulated secretion of VWF suggesting that V-ATPase mediated acidification is not required for WPB exocytosis [12, 22]. However, the VWF filaments released from neutralized WPB are shorter and/or more tangled and less efficient in platelet recruitment [12] suggesting that the compaction and tubulation of VWF inside acidic WPB is important for its biological activity as platelet receptor after secretion into the circulation.

**Fig 5:**
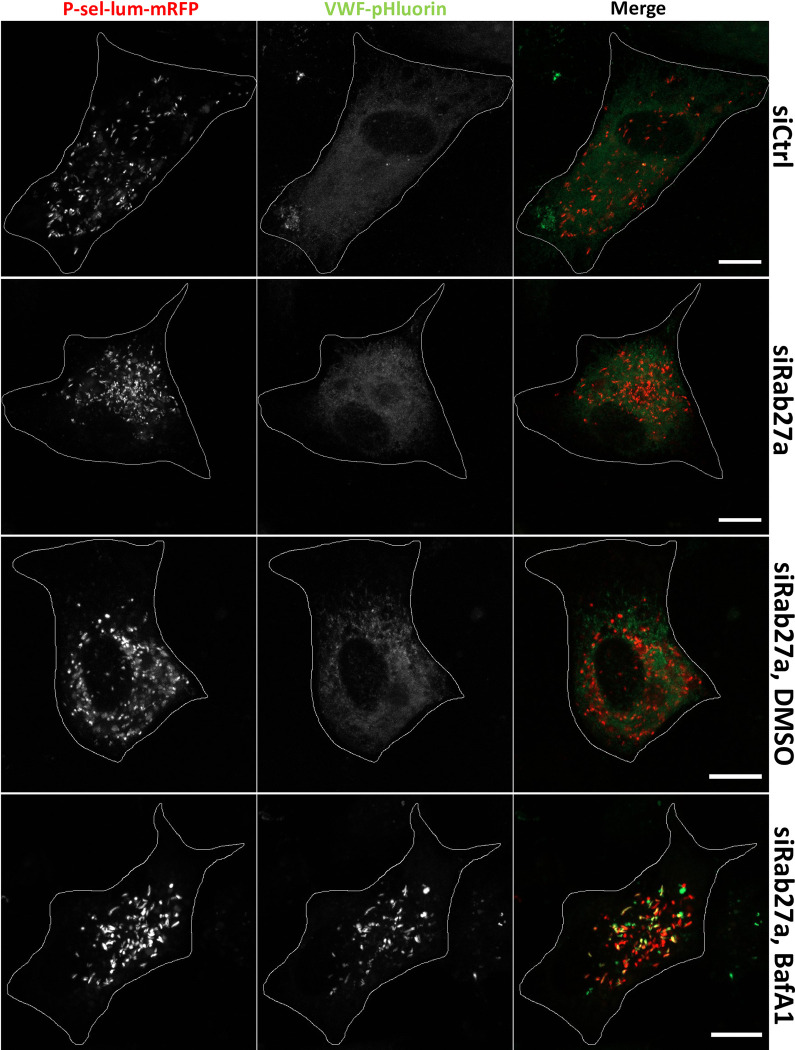
Rab27a is not involved in acidification of WPB. HUVEC were transfected with untargeting siRNA (siControl) or siRNA directed against Rab27a, both together with P-sel-lum-mRFP and VWF-pHluorin. After 24 h, cells were analyzed directly via confocal live cell microscopy or incubated with 0.1% DMSO or 250 nM BafA1, respectively, at 37°C for 2 h followed by live cell imaging. Shown are images of maximum intensity projections of z-stacks. Cell circumferences are indicated by lines. Scale bars: 10 μm.

The finding that V-ATPase inhibition/depletion interferes with proper WPB maturation is also supported by a closer morphological inspection of the VWF-pHluorin positive WPB in BafA1 and archalzolid treated HUVEC and also in cells depleted of V0d1. They appear less elongated (Figs 2–4; quantified for BafA1 treated cells in S4 Fig), a phenotype also seen when proper WPB maturation is inhibited, e.g. by Rab27a and MyRIP depletion [34]. Given this phenotype and the fact that V-ATPase subunits were identified in the Rab27a proximity proteome, we next analyzed whether Rab27a is involved in regulating V-ATPase activity in WPB. This appeared plausible as Rab27a had been shown before to target several effectors to maturing WPB, e.g. MyRIP and Slp4-a [34, 35]. Therefore, HUVEC were depleted of Rab27a by an established siRNA treatment [36] and then subjected to the VWF-pHluorin/P-sel-lum-mRFP fluorescence microscopy assay. As seen for wildtype cells, the VWF-pHluorin signal was not visible in the P-sel-lum-mRFP positive WPB in control and vehicle treated cells and increased significantly upon BafA1 incubation (Fig 5). Thus, although the effects of V-ATPase inhibition were particularly visible in mature WPB and this inhibition altered the morphological appearance of WPB, Rab27a itself was not responsible for mediating these effects and hence, an association of the V-ATPase with WPB.

The V1 and V0 sectors of the V-ATPase can assemble or disassemble upon various stimuli. For example, in mammalian cells assembly on lysosomes can be triggered by high glucose concentrations, viral infections, lysosome neutralization or amino acid starvation (reviewed by [25, 31]). However, how and when association of the V-ATPase sectors on WPB occurs is not known and future experiments have to address this issue. Future studies should also clarify whether organelle-specific subunit(s) define the V-ATPase on WPB. In fact, several V-ATPase isoforms are expressed in a tissue specific manner and a differential subcellular targeting of V-ATPase a-subunits was described before [37, 38] (for reviews see [39, 40]). Recently it was shown that the subunit V0a2 is present on perinuclear WPB [22] suggesting that V-ATPase complexes positive for this subunit could originate from a pool of V-ATPases at the TGN. On the other hand, the subunit V0a1 appears to localize to peripheral WPB. This switch of a-subunits could be due to additional trafficking pathways of V-ATPase subunits to WPB. In line with this assumption Ma et al. described misshaped WPB, similar to the WPB seen NH_4_Cl-treated cells, upon the depletion of HPS6, a subunit of the endosomal BLOC-2 complex, suggesting that endosomes could serve as a further potential source of V-ATPase complexes for WPB [41]. This view is supported by the recently identified direct interaction between HPS6 and the V0d1 subunit [33].

## Material and methods

### Cell culture and transfection

HUVEC were acquired from PromoCell as cryoconserved pools (C-12203) and cultured on Corning CellBind dishes at 37°C and 5% CO_2_ in 1:1 mixed medium comprising M199 medium (BioChrom) supplemented with 10% FCS, 30 μg/ml gentamycin, 0.015 μg/ml amphotericin B, and ECGMII (PromoCell) supplemented with 30 μg/ml gentamycin, 0.015 μg/ml amphotericin B. Experiments were conducted with HUVEC passage 3–5.

HUVEC were transfected using the Amaxa nucleofection system (Lonza) according to the manufacturer´s specifications. Per cuvette, cells from a confluent 20–30 cm^2^ dish together with 1–6 μg plasmid DNA and/or 400 pmol siRNA were resuspended in transfection buffer (4 mM KCl, 10 mM MgCl_2_, 10 mM sodium succinate, 100 mM NaH_2_PO_4_, pH 7.4 adjusted with NaOH). For depletion of proteins using siRNA, HUVEC were transfected with 400 pmol of siRNA twice. 48 h after the first transfection the cells were transfected again with the same amount of the respective siRNA. 24 h after the second transfection cells were subjected to live cell microscopy or lysed for Western blot analysis.

For live cell microscopy, transfected HUVEC were seeded on 8-chamber μ-slides which were freshly coated with collagen from rat tail (Advanced Biomatrix, 5056) at a concentration of 50 μg/ml in 0.02 M acetic acid solution. Right before imaging confluent HUVEC, the mixed medium was exchanged with Hank´s Balanced Salt Solution (Sigma, H6648), herein referred to as HBSS, supplemented with 20 mM HEPES pH 7.0–7.6 (Sigma, H0887).

### Plasmids and siRNA

A P-selectin construct encoding only the soluble luminal domain of P-selectin, herein referred to as P-sel-lum-mRFP was subcloned from P-selectinLum-mCherry, which was kindly provided by Dan Cutler (MRC Laboratory for Molecular Cell Biology, University College London) [42, 43], by introducing the P-selectinLum coding region into the pmRFP1-N1 vector from Addgene (Plasmid No: 54635) via BglII/AgeI restriction sites (courtesy of Nina Criado Santos, Institute of Medical Biochemistry, University of Muenster).

VWF-pHluorin was obtained by replacing mRFP in a VWF-mRFP plasmid, which was kindly provided by Tom Carter (St. George´s University of London, UK) [44, 45], with superecliptic pHluorin [46] (courtesy of Anja Biesemann, Institute of Medical Biochemistry, University of Muenster).

Rab27a-EGFP was generated by amplification of the Rab27a cDNA from a HUVEC cDNA library using specific primers that included EcoRI and BamHI restriction sites at the 5’ and 3’ end, respectively, and cloning of the PCR product into the pEGFP-C1 vector (Clontech). Rab27a-mStrawberry was then generated by exchanging the fluorophore of Rab27a-EGFP, using pmStrawberry (Clontech) as the fluorophore source and specific primers with the restriction sites AgeI and BamHI at the 5’ and 3’ end, respectively.

VWF-pHluorin-mRFP was obtained by amplification of VWF-pHluorin (see above) with the following primers: 5´- GTTGCTAGCCTCGAGCTCAAGCTTCGGATTCATGATTC and 5´- CTTGAATTCCGCGGCCGCTTGATTTGTATAGT. The PCR product was then inserted into the pmRFP1-N1 vector from Addgene (Plasmid No: 54635) via NheI and EcoRI restriction sites.

siRNA targeting ATP6V0d1 was obtained from Horizon Discovery (L-019238-02-0010). Rab27a was depleted using the siRNA 5`GGAGAGGUUUCGUAGCUUA-dTdT purchased from Microsynth [36]. AllStars Negative Control siRNA was from Qiagen (102781).

### Antibodies

For imaging, mouse monoclonal anti-VWF antibodies were acquired from DAKO (M061601-2) and rabbit polyclonal anti-ATP6V1G1 antibodies (16143-1-AP) were obtained from Proteintech. Secondary antibodies (AlexaFluor488, AlexaFluor594) were purchased from Molecular Probes. Rabbit polyclonal anti-ATP6V0d1 (Proteintech, 8274-1-AP), mouse monoclonal anti-α-tubulin clone B-5-1-2 (Sigma-Aldrich T5168), rabbit monoclonal anti-α-tubulin clone 11H10 (Cell Signaling Technology 2125), mouse monoclonal anti-RFP (Chromotek 6G6) and rabbit polyclonal anti-GFP (Chromotek PABG1) antibodies were used as primary antibodies in Western blot analysis.

### Western blot analysis

For preparation of cell lysates, HUVEC were harvested using trypsin/EDTA. After washing once with PBS, cell pellets were resuspended in 30 μl RIPA buffer (25 mM Tris-HCl, 150 mM NaCl, 0.1% (w/v) SDS, 0.5% (w/v) sodium deoxycholate, 1% (v/v) Triton X-100, pH 7.5) supplemented with 1x Complete protease inhibitor cocktail (Roche, 04693116001) per 20 cm^2^ confluent HUVEC and lysed for 5 min on ice followed by 1 min sonification and 15 min prolonged lysis on ice. Cellular debris was removed by centrifugation at 1250 x g for 10 min at 4°C. For deglycosylation, 10 μl PNGase F (Promega, V483A) was added to the supernatant and the sample was incubated for 3 h at 37°C. Protein loading buffer was added to a final concentration of 1x and the samples were incubated for 10 min at 95°C.

Samples were subjected to 10% or 8% SDS-PAGE for 30 min at 70 V and subsequently at 120 V, and blotted onto 0.2 μm nitrocellulose membrane in a wet tank system at 115 V for 1h at 4°C in Tris-Glycine buffer (25 mM Tris, 190 mM glycine, 20% (v/v) methanol). Membranes were blocked using 3% skim milk in TBST (150 mM NaCl, 20 mM Tris-HCl, 0.1% (v/v) Tween-20, pH 7.4) for at least 30 min and incubated with primary antibodies over night at 4°C. For signal detection, infrared conjugated secondary antibodies (IRdye680RD or IRdye800CW, LICOR) and the Odyssey imaging system (LICOR) were used.

### Experiments with HUVEC expressing VWF-pHluorin and P-sel-lum-mRFP or VWF-pHluorin-mRFP

Bafilomycin A1 (BafA1) was purchased from Cayman Chemical (Cay11038-1) and dissolved in DMSO. Aliquots of 250 μM were stored at -20°C. Archazolid A (ArchA) was dissolved in DMSO and aliquots of 10 μM were stored at -20°C.

24 h after seeding on 8-chamber μ-slides, transfected HUVEC were incubated with media containing 250 nM BafA1 for 2 h, 0.1% DMSO for 3 h or 40 mM NH_4_Cl for 3 h at 37°C. Subsequently medium was exchanged with HBSS, supplemented with 20 mM HEPES pH 7.0–7.6 and containing 250 mM BafA1, 0.1% DMSO or 40 mM NH_4_Cl, respectively, and cells were subjected to live cell imaging at 37°C for less than 1 h.

For treatment with ArchA, cells were grown for 24 h on 8-chamber μ-slides following incubation with 2 nM ArchA or 0.02% DMSO for 24 h [47]. Subsequently, medium was exchanged with HBSS supplemented with 20 mM HEPES, pH 7.4, and containing 2 nM ArchA or 0.02% DMSO, respectively, and cells were subjected to live cell imaging at 37°C.

For imaging of HUVEC subjected to siRNA treatment (see above), cells were additionally transfected with P-sel-lum-mRFP and VWF-pHluorin together with the second round of siRNA transfection and seeded on 6 cm dishes or 8-chamber μ-slides. Approximately 24 h after the second transfection, cells were lysed for Western blot analysis or analyzed via live cell microscopy at 37°C.

### Determination of the Feret diameter of WPB

HUVEC cotransfected with P-sel-lum-mRFP and VWF-pHluorin were incubated with 0.1% DMSO or 250 nM BafA1, respectively, at 37°C for 2 h and subsequently subjected to live cell imaging. The Feret diameter of WPB was obtained by analyzing a single confocal plane of an imaged cell using ImageJ. For HUVEC treated with BafA1 the Feret diameter was determined from the pHluorin signal of 893 neutralized WPB (10 cells). For HUVEC treated with DMSO the Feret diameter of 1217 WPB (10 cells) was determined using the mRFP signal.

### Immunofluorescence stainings

Cells were cultivated on collagen coated coverslips (12 mm diameter) until they reached confluency, then fixed in 4% PFA in PBS for 10 min at RT and permeabilized using 0.1% Triton X-100 in PBS for 2 min. Unspecific binding was blocked by addition of 3% BSA in PBS for at least 30 min, followed by antibody incubation over night at 4°C in 3% BSA in PBS. Secondary antibodies were diluted 1:200 and incubated for 40 min at room temperature (RT). DAPI staining was conducted with a 0.1 μg/ml solution (Sigma, D9542) for 10 min at RT. After extensive washing, samples were mounted in mounting medium.

### Microscopy

Confocal microscopy was performed using an LSM 800 microscope (Carl Zeiss) equipped with a Plan-Apochromat 63×/1.4 oil immersion objective.

During live cell microscopy pHluorin was excited at 488 nm and emitted light of 490–550 nm was detected. mRFP was excited by the 561 nm laser line and emitted light of 580–700 nm was detected. For fixed samples stained with AlexaFluor488 and AlexaFluor594 excitation was conducted at 488 nm and 561 nm and emitted light of 490–580 nm and 580–700 nm was detected.

### Image analysis

Confocal images were analyzed using ImageJ. Colocalization analysis was performed by using the plugin JACoP [48]. The extent of cooccurrence of mRFP with pHluorin was quantified by calculating Manders coefficient 1 which determines the percentage of total signal from mRFP overlapping with signal from pHluorin [49, 50].

### Statistics

All statistics were performed using GraphPad PRISM. Asterisks mark statistically significant results: ****p ≤ 0.0001, ***p ≤ 0.001, **p ≤ 0.01, *p ≤ 0.05. Normal distribution was assessed by the Shapiro-Wilk-Test, p < 0.05. Normally distributed data was analyzed employing an unpaired t-test with Welch´s correction. Non-parametric data was analyzed using a Mann-Whitney test.

## Supporting information

S1 Fig Expression of VWF-pHluorin and P-sel-lum-mRFP in HUVEC. HUVEC were lysed 24 h after transfection with P-sel-lum-mRFP or VWF-phluorin and lysates were subjected to SDS-PAGE followed by Western blotting with anti-GFP or anti-RFP antibodies. PNGase F treatment for 3 h was used to deglycosylate P-sel-lum-mRFP. The rightmost lanes in each blot show lysates from cells transfected with control GFP or mRFP plasmids, respectively. Probing with anti-tubulin antibodies served as loading control (bottom). The raw data is shown in S1 Raw images.
(TIF)

S2 Fig VWF-pHluorin acts as an intraluminal pH-indicator for WPB. HUVEC cotransfected with P-sel-lum-mRFP and VWF-phluorin were incubated with 40 mM NH_4_Cl for 3 h and then analyzed by live cell confocal microscopy. Shown are images of maximum intensity projections of z-stacks. Cell circumferences are indicated by lines. Scale bar: 10 μm.
(TIF)

S3 Fig VWF-pHluorin-mRFP reports neutralization of WPB upon treatment with bafilomycin A1. **A.** Colocalization of fluorescence signals emitted by pHluorin and mRFP both fused in tandem to VWF in HUVEC treated with the V-ATPase-specific inhibitor bafilomycin A1. HUVEC transfected with VWF-pHluorin-mRFP were incubated with 0.1% DMSO (upper panel) or 250 nM BafA1 (lower panel), respectively, at 37°C for 2 h and subsequently subjected to live cell imaging. Shown are images of maximum intensity projections of z-stacks. Cell circumferences are indicated by lines. Scale bars: 10 μm. **B.** The degree of colocalization of the fluorescence signals was quantified by determining Manders coefficient 1 which corresponds to the fraction of mRFP fluorescence overlapping with pHluorin fluorescence. n = 10. Bars indicate the median. Error bars show 95% confidence interval. Statistics were conducted using a Mann-Whitney test. ****p≤ 0.0001, ***p≤ 0.001, **p≤0.01, *p≤0.05.
(TIF)

S4 Fig The elongated shape of WPB is affected by treatment with BafA1. HUVEC cotransfected with P-sel-lum-mRFP and VWF-pHluorin were incubated with 0.1% DMSO (shown in grey) or 250 nM BafA1 (shown in black), respectively, at 37°C for 2 h and subsequently subjected to live cell imaging. For HUVEC treated with BafA1 the Feret diameter was determined from the pHluorin signal of 893 neutralized WPB (10 cells). For HUVEC treated with DMSO the Feret diameter of 1217 WPB (10 cells) was determined using the mRFP signal.
(TIF)

S1 Raw images (TIF)
