# Supplementary figures and images for: Acidification of endothelial Weibel-Palade bodies is mediated by the vacuolar-type H+-ATPase
Source: PLoS One. 2022 Jun 29;17(6):e0270299. doi: 10.1371/journal.pone.0270299 (PMC9242466; doi:10.1371/journal.pone.0270299)

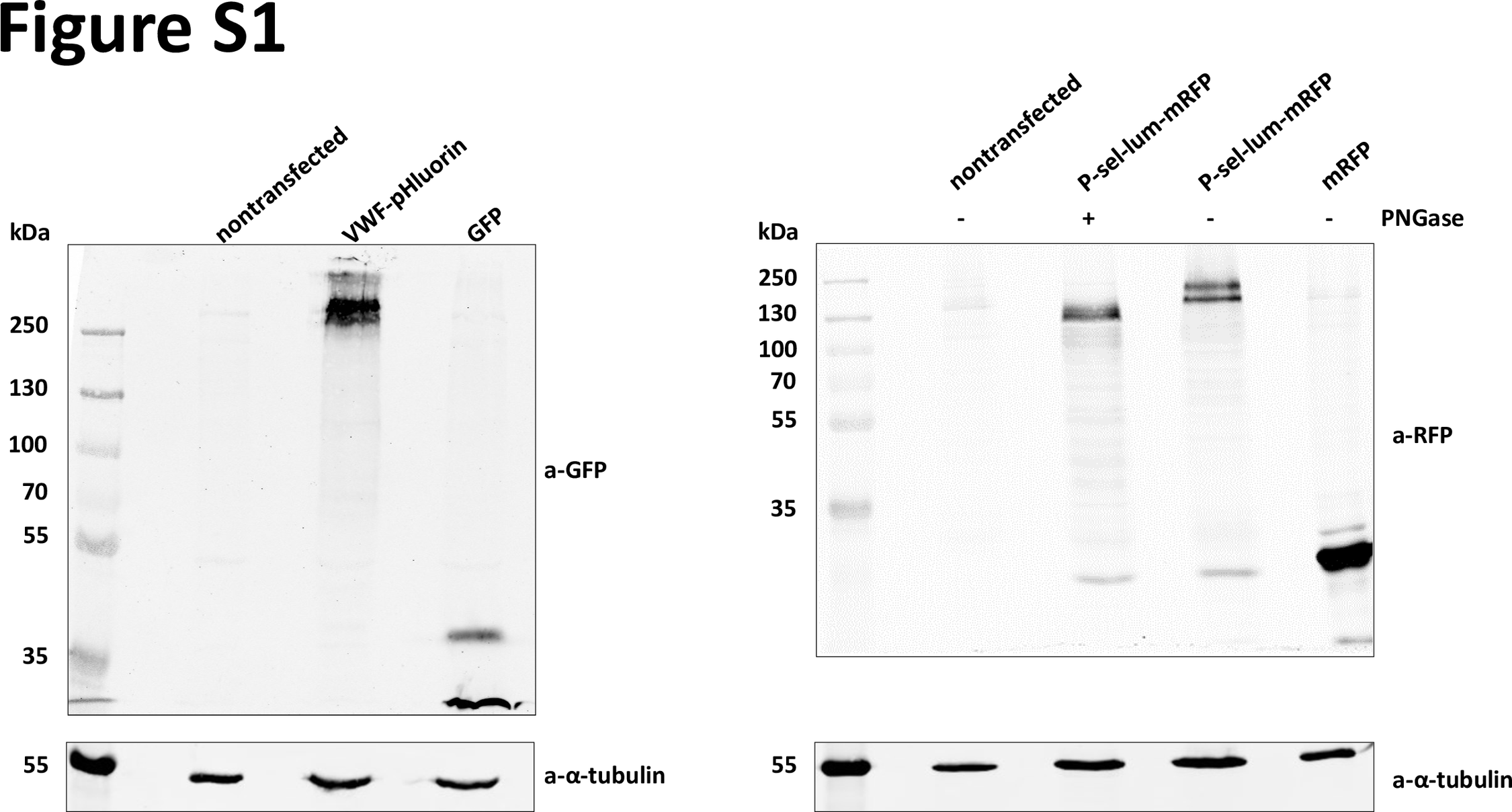

Supplement: S1 Fig — HUVEC were lysed 24 h after transfection with P-sel-lum-mRFP or VWF-phluorin and lysates were subjected to SDS-PAGE followed by Western blotting with anti-GFP or anti-RFP antibodies. PNGase F treatment for 3 h was used to deglycosylate P-sel-lum-mRFP. The rightmost lanes in each blot show lysates from cells transfected with control GFP or mRFP plasmids, respectively. Probing with anti-tubulin antibodies served as loading control (bottom). The raw data is shown in S1 Raw images. (TIF) [file pone.0270299.s001.tif]

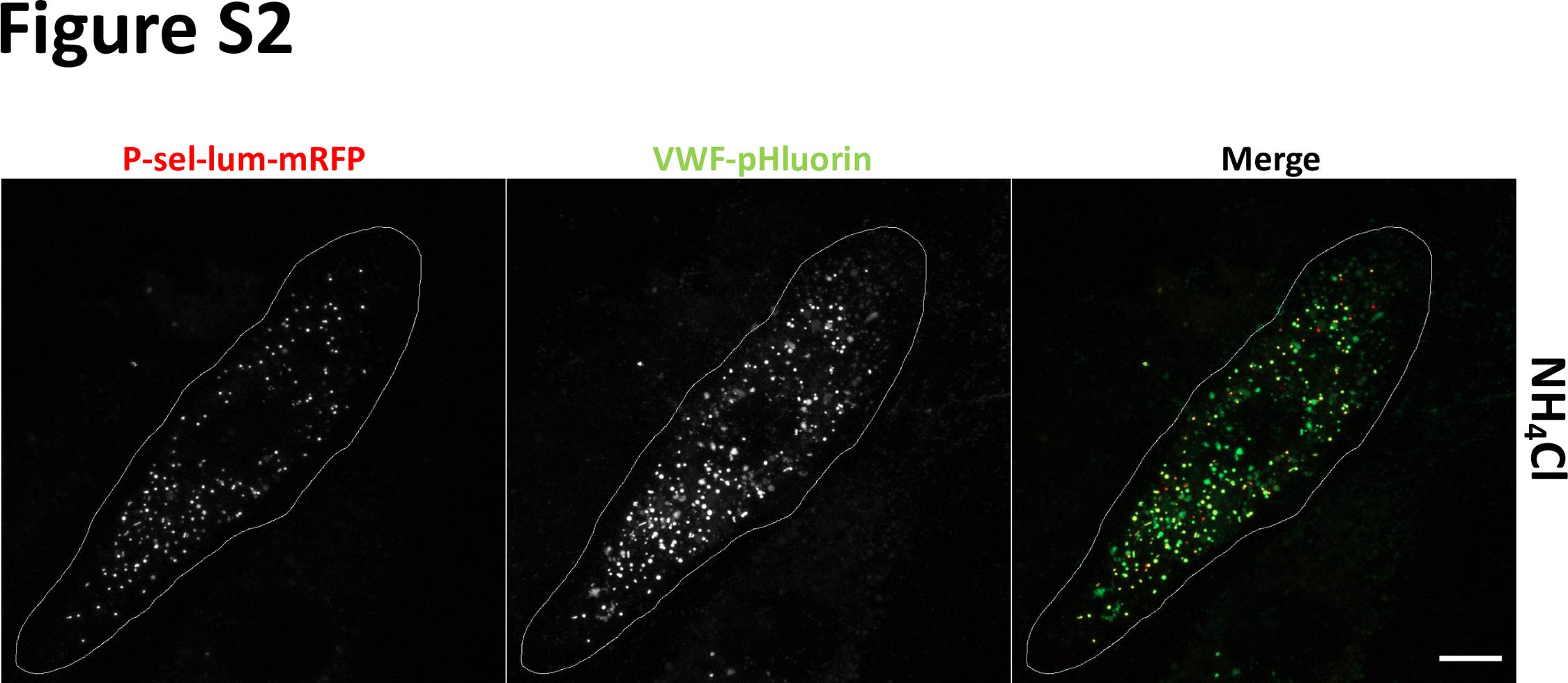

Supplement: S2 Fig — HUVEC cotransfected with P-sel-lum-mRFP and VWF-phluorin were incubated with 40 mM NH4Cl for 3 h and then analyzed by live cell confocal microscopy. Shown are images of maximum intensity projections of z-stacks. Cell circumferences are indicated by lines. Scale bar: 10 μm. (TIF) [file pone.0270299.s002.tif]

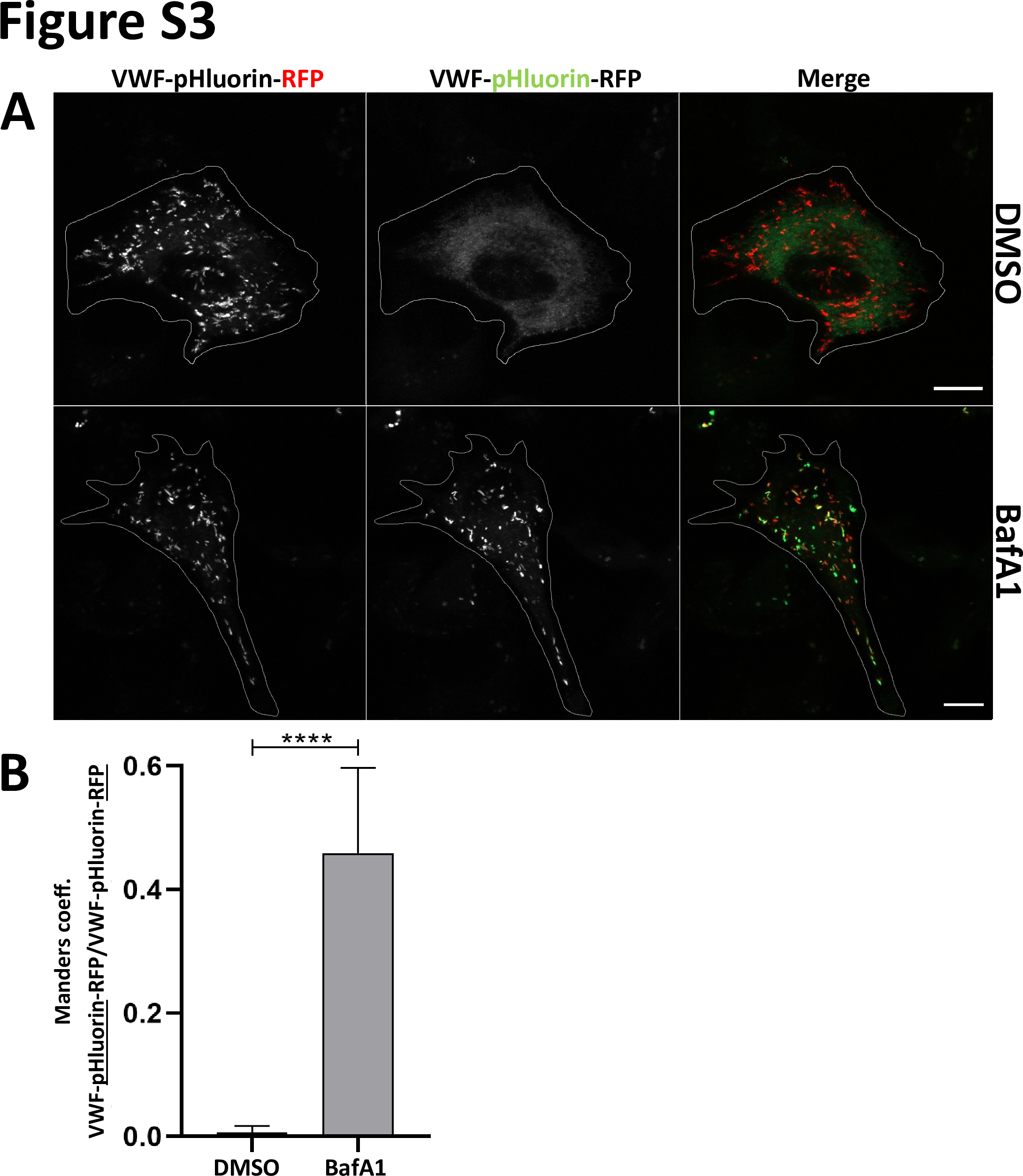

Supplement: S3 Fig — A. Colocalization of fluorescence signals emitted by pHluorin and mRFP both fused in tandem to VWF in HUVEC treated with the V-ATPase-specific inhibitor bafilomycin A1. HUVEC transfected with VWF-pHluorin-mRFP were incubated with 0.1% DMSO (upper panel) or 250 nM BafA1 (lower panel), respectively, at 37°C for 2 h and subsequently subjected to live cell imaging. Shown are images of maximum intensity projections of z-stacks. Cell circumferences are indicated by lines. Scale bars: 10 μm. B. The degree of colocalization of the fluorescence signals was quantified by determining Manders coefficient 1 which corresponds to the fraction of mRFP fluorescence overlapping with pHluorin fluorescence. n = 10. Bars indicate the median. Error bars show 95% confidence interval. Statistics were conducted using a Mann-Whitney test. ****p≤ 0.0001, ***p≤ 0.001, **p≤0.01, *p≤0.05. (TIF) [file pone.0270299.s003.tif]

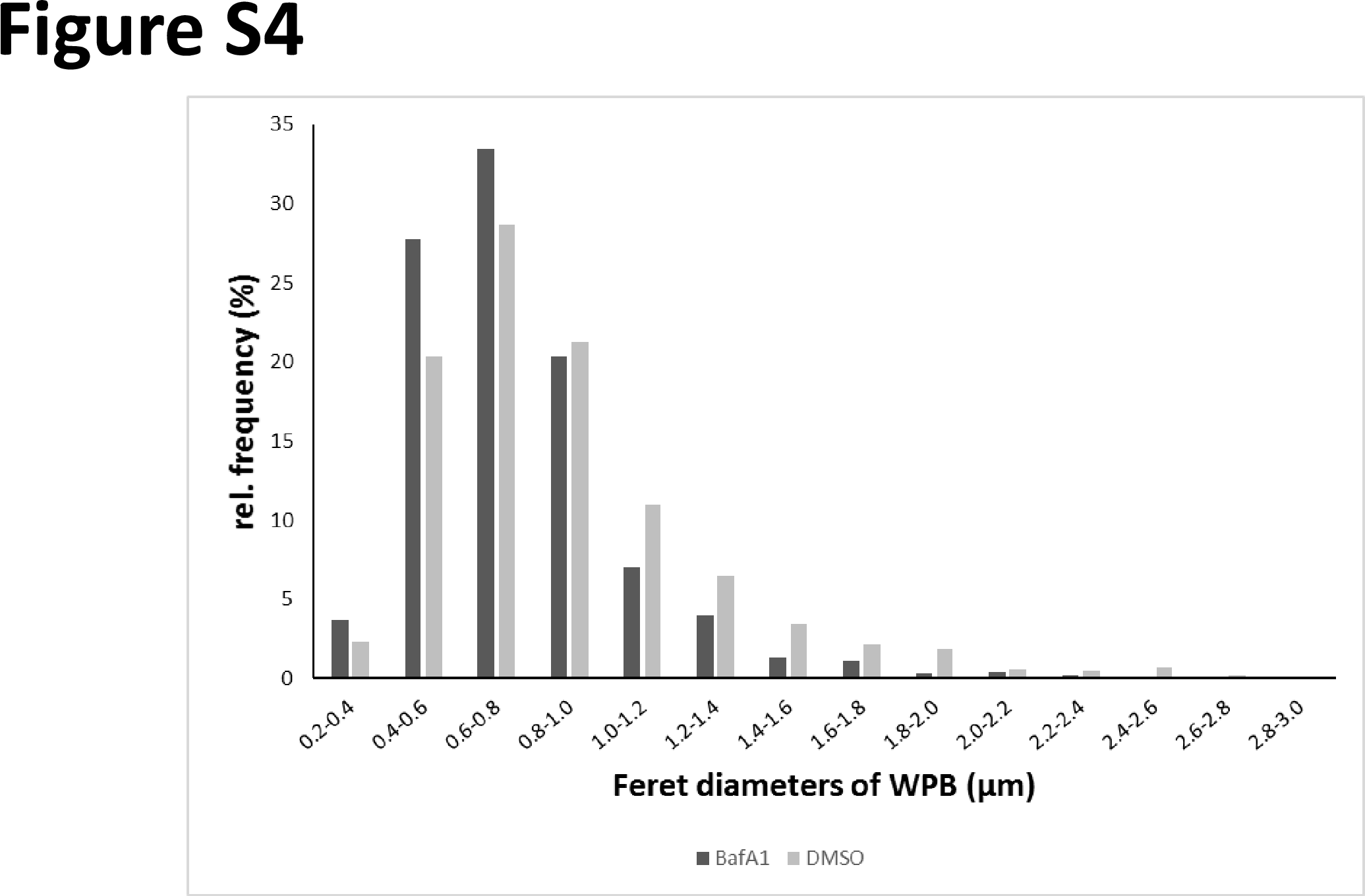

Supplement: S4 Fig — HUVEC cotransfected with P-sel-lum-mRFP and VWF-pHluorin were incubated with 0.1% DMSO (shown in grey) or 250 nM BafA1 (shown in black), respectively, at 37°C for 2 h and subsequently subjected to live cell imaging. For HUVEC treated with BafA1 the Feret diameter was determined from the pHluorin signal of 893 neutralized WPB (10 cells). For HUVEC treated with DMSO the Feret diameter of 1217 WPB (10 cells) was determined using the mRFP signal. (TIF) [file pone.0270299.s004.tif]

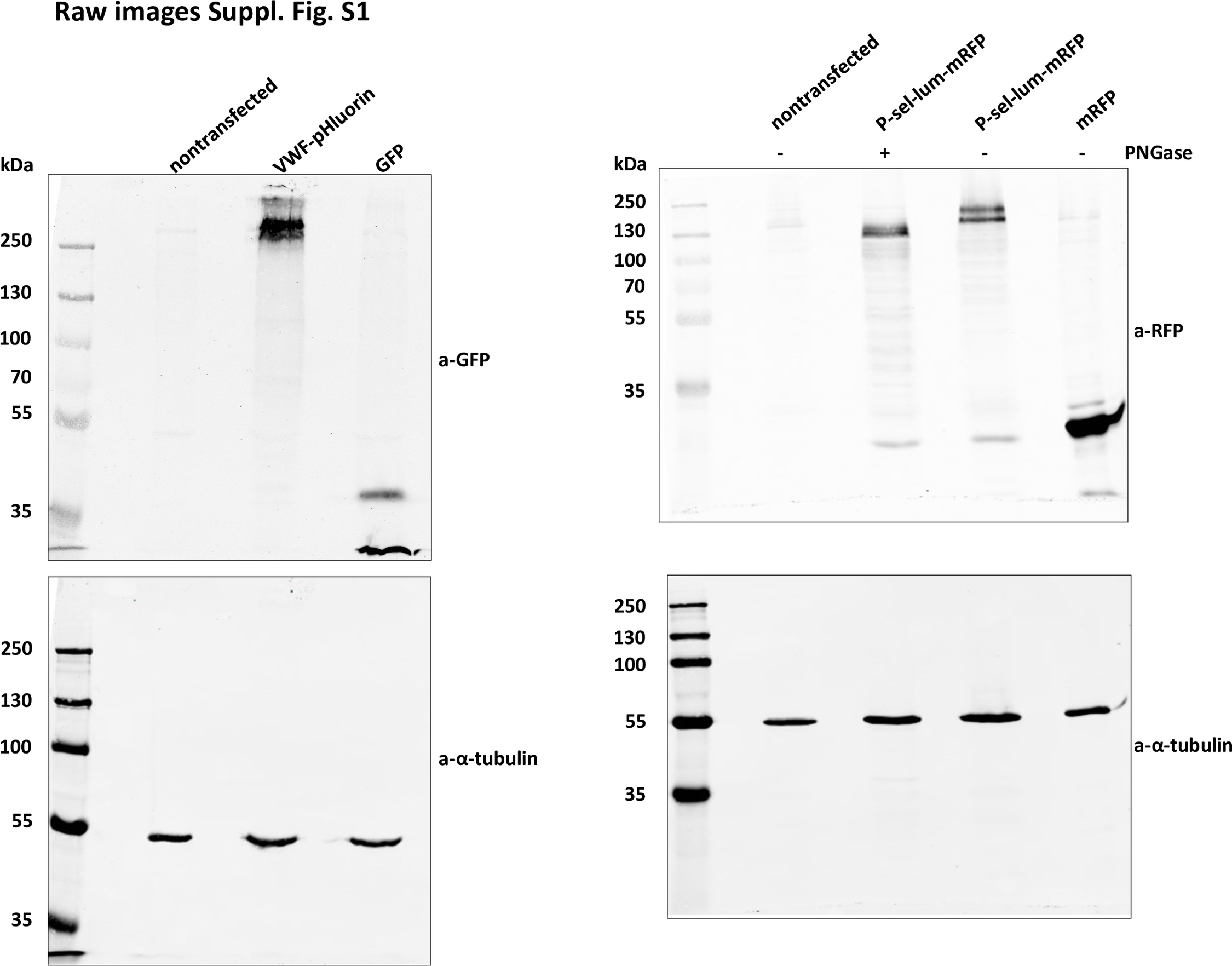

Supplement: S1 Raw images — (TIF) [file pone.0270299.s005.tif]
